# Safety and efficacy of direct oral anticoagulants compared to Vitamin K antagonists postpercutaneous coronary interventions in patients with atrial fibrillation: A systematic review and meta‐analysis

**DOI:** 10.1002/joa3.12292

**Published:** 2020-01-08

**Authors:** Pradyumna Agasthi, Justin Z. Lee, Sai Harika Pujari, Andrew S. Tseng, Justin Shipman, Diana Almader‐Douglas, Hasan Ashraf, Farouk Mookadam, Floyd David Fortuin, Nirat Beohar, Reza Arsanjani, Siva Mulpuru

**Affiliations:** ^1^ Department of Cardiovascular Diseases Mayo Clinic Phoenix AZ USA; ^2^ Department of Cardiovascular Diseases Mayo Clinic Rochester MN USA; ^3^ Library Services Mayo Clinic Phoenix AZ USA; ^4^ Department of Cardiovascular Diseases Mount Sinai Medical Center Miami FL USA

**Keywords:** atrial fibrillation, direct oral anticoagulants, percutaneous coronary intervention, vitamin K antagonists

## Abstract

**Background:**

Atrial fibrillation (AF) and coronary artery disease (CAD) are commonly associated. Cotreatment with multiple antithrombotic agents can increase the risk of bleeding. We sought to evaluate patient‐centered outcomes in patients with AF on double therapy with direct oral anticoagulants (DOACs) compared to patients with standard triple therapy, [a vitamin K antagonist (VKA) plus dual antiplatelet therapy].

**Methods:**

We performed a literature search of randomized controlled trials (RCTs) reporting outcomes of patients receiving double therapy with DOACs compared to triple therapy with VKAs in patients with AF undergoing percutaneous coronary intervention (PCI). Patient‐centered outcomes were the International Society of Thrombosis and Hemostasis (ISTH) major or clinically relevant nonmajor bleeding (CRNB), all‐cause mortality, major adverse cardiovascular events (MACE), stent thrombosis, myocardial infarction, and stroke.

**Results:**

Four RCTs (9602 patients) met our inclusion criteria. Compared to VKAs, DOACs were associated with significantly lower ISTH major bleeding/ CRNB (RR: 0.75, 95% CI: 0.67‐0.82, *P* < .00001, *I* ^2^ = 11%). There were no statistically significant differences in the efficacy outcomes, including myocardial infarction (RR: 0.99, 95% CI :0.79‐1.25, *P* = .96), stent thrombosis (RR: 0.97, 95% CI: 0.6‐1.55, *P* = .89), ischemic stroke (RR: 0.76, 95% CI: 0.5‐1.15, *P* = .19), all‐cause mortality (RR: 1.06, 95% CI: 0.85‐1.31, *P* = .61), and MACE (RR: 1.06, 95% CI: 0.91‐1.22, *P* = .97).

**Conclusion:**

Compared with triple therapy with VKAS, double therapy with DOACs is associated with a reduced risk of bleeding and is as effective in patients with AF undergoing PCI.

## Graphical abstract

In our meta-analysis incorporating 4 randomized control trials with 9602 patients, triple therapy with Vitamin K antagonists compared to double therapy with DOACs is associated with a reduced risk of bleeding and is as effective in patients with atrial fibrillation undergoing percutaneous coronary intervention.

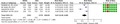

## 1 INTRODUCTION

One of the common comorbidities of coronary artery disease (CAD) is atrial fibrillation (AF).^1^, ^2^, ^3^ Its prevalence is about 2% in the general population and increases with age.^2^ Inflammation plays an important role in the development of both conditions, and they share associated risk factors, including diabetes mellitus, hypertension, sleep apnea, obesity, and smoking.^1^, ^2^, ^4^, ^5^, ^6^ Up to 30% of AF patients have concomitant CAD, of whom 5%‐10% are PCI patients.^7^, ^8^ The management of CAD and AF is distinct, as anticoagulants are used in AF, and antiplatelet drugs are used in CAD. Hence, combination of antithrombotic therapy with anticoagulants and antiplatelet drugs may lead to excessive bleeding and result in serious complications.^1^ These combinations include double therapy (an oral anticoagulant plus a P2Y12 inhibitor) or triple therapy (an oral anticoagulant plus dual antiplatelet therapy).

Over the past few decades, anticoagulation options have expanded rapidly, offering a wider amount of agents for thromboembolic disease prevention and management.^9^ No anticoagulant can reduce the risk of thrombosis without increasing the risk of bleeding to a certain extent. The emergence of direct oral anticoagulants (DOACs) has completely reshaped the management of AF.^1^, ^10^, ^11^, ^12^, ^13^, ^14^, ^15^ The current American and European professional society guidelines recommend DOACs as the first‐line treatment in AF.^16^, ^17^ Nevertheless, vitamin K antagonists (VKAs) are recommended when combined with dual antiplatelet therapy (DAPT).^18^, ^19^

This meta‐analysis compares the safety and efficacy outcomes for four randomized controlled trials (RCTs) on double therapy with DOACs vs standard triple therapy with VKAs in AF and PCI. Previous meta‐analyses assessed the safety and efficacy of DOACs in patients with AF who undergo PCI with comparing PIONEER AF‐PCI and RE‐DUAL PCI trials.^1^ The present analysis compares two more recent trials (AUGUSTUS and ENTRUST) to assess the safety and efficacy of DOACs in patients with AF who have undergone PCI.

## 2 METHODS

### 2.1 Protocol and registration

The protocol detailing the methods of the systematic review and meta‐analysis was registered on the International Prospective Register of Systematic Reviews. The current meta‐analysis was performed using the guidelines set by the Preferred Reporting Items for Systematic Reviews and Meta‐Analyses (PRISMA).^20^ Given the nature of the study, the meta‐analysis was exempted from institutional review.

### 2.2 Study identification and search strategy

We performed a search for RCTs using OVID versions of Medline (2000‐2019), EMBASE (2000‐2019), SCOPUS (1999‐current), Web of Science (2000‐2019), and Cochrane Database (2001‐2019). The authors (PA and JZL) developed the search strategy working with a clinical information specialist (DA –D.). The last search was run on October 4, 2019. Details of the search strategy are provided in the Data S1.

### 2.3 Study selection

Two reviewers (PA and JZL) performed initial screening of the search results for inclusion into the meta‐analysis. The first step involved title and abstract screening. The second step involved comprehensive review of the entire manuscripts. Inconsistencies in screening were resolved with consensus or when consensus could not be achieved, a third reviewer (JS) casted the deciding vote.

### 2.4 Eligibility criteria

We selected all published RCTs, including any adult (age > 18 years) population with AF who underwent PCI comparing double therapy with DOAC to triple therapy with VKA following PCI. All classes of DOAC were included. No restrictions on study selection based on outcomes were used.

### 2.5 Data extraction and quality assessment

For each included study, we extracted: (a) Characteristics of study participants including age, gender, race, type of AF, index event, medical history, type of stent, antiplatelet at randomization, type of oral anticoagulant before PCI, and the study's inclusion and exclusion criteria; (b) types of intervention DOACs (apixaban, edoxaban, dabigatran, and rivaroxaban) vs VKAs; and (c) outcome measures including ISTH major or clinically relevant nonmajor bleeding, all‐cause mortality, major adverse cardiovascular events, stroke, myocardial infarction, or stent thrombosis.

A standardized data extraction sheet for study screening based on the Cochrane Consumers and Communication Review Group's data extraction template was developed. Two randomly selected included studies were piloted on the extraction sheet and adjustments were made accordingly. The two authors independently collected the data and agreement measures were reported using Kappa values.

### 2.6 Risk of bias

Validity of eligible RCTs was ascertained by pairs of reviewers, independently and determined the adequacy of concealment of allocation and randomization, blinding of patients, collectors, data, outcome assessors, health care providers, and extent of loss to follow‐up (ie, proportion of patients in whom the investigators were not able to ascertain outcomes). To explore heterogeneity (variability) in study results, the hypotheses that effect size may vary according to the quality of RCTs were specified before performing the analysis. For each study, the effect by inverse of the standard error was plotted for each study. The assessment of symmetry of “funnel plots” was performed visually.

### 2.7 Method of analysis

The meta‐analysis was performed by computing risk ratios (RRs) using the random effects model based on underlying statistical heterogeneity. We calculated the RR and 95% confidence intervals (CIs) for each treatment effect for each study and combined them using Review Manager Version 5.3. (Copenhagen: The Nordic Cochrane Centre, The Cochrane Collaboration, 2014). We tested statistical heterogeneity using the *I* ^2^ statistics. The *I* ^2^ statistics describe the percentage of variation across studies that is because of heterogeneity rather than those expected by random chance [*I* ^2^ = 100% × (Q‐*df*)/Q]. A CI for *I* ^2^ is constructed using either (a) the iterative noncentral chi‐squared distribution method of Hedges and Piggott (2001) or (b) the test‐based method of Higgins and Thompson. We created summary of evidence table to summarize the main results (patient‐centered outcomes) of the meta‐analysis using GRADE Pro tool (Guideline Development Tool [Software], McMaster University, 2015 [developed by Evidence Prime, Inc]).^21^

## 3 RESULTS

### 3.1 Study selection

**Figure 1:**
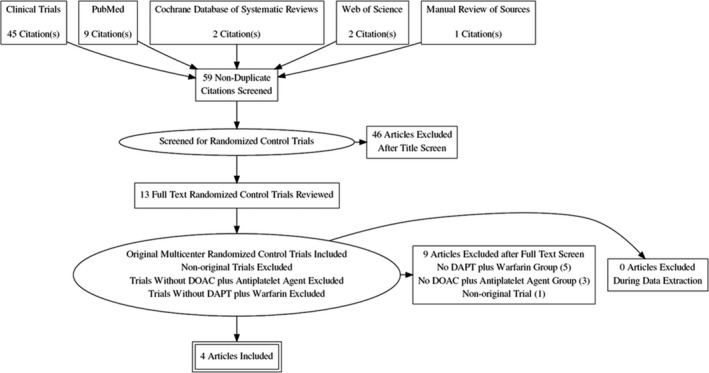
PRISMA flow chart of the RCT selection for the meta‐analysis

A total of 59 Citations were identified using Medline, EMBASE, SCOPUS, Web of Science, and Cochrane databases. A total of four RCTs were selected to be included in the evaluation.^22^, ^23^, ^24^, ^25^ Based on the title and abstracts, 46 studies were excluded. The rest of the publications were studied in detail, and four studies met the inclusion criteria mentioned above. The PRISMA diagram for the systematic review is shown in Figure 1. Kappa for agreement on full text, and abstract inclusion was 0.89 (95% CI: 0.86‐0.94).

### 3.2 Study and patient characteristics

The study characteristics are summarized in Table 1. The included trials were published between 2016 and 2019. All of the trials were multi‐centered and had a follow‐up duration of 6 months to 12 months. The baseline characteristics of each RCT are provided in the Data S1. A total of 6733 patients were included in this meta‐analysis, with the sample size in each trial ranging from 1393 to 2307. The mean age of patients was between 68 and 70 years. The patients were predominantly white (91.7%‐94.1%), and ACS prevalence ranged from 30% to 52%. Comorbidities like diabetes, hypertension, hypercholesterolemia, and peripheral artery disease, ranged from 5% to 88%. Most of the patients had a CHA2DS2‐VASc score > 3 and a HAS‐BLED score > 2.5. All patients had undergone PCI, except for AUGUSTUS, in which patients have had an acute coronary syndrome or have undergone PCI. Time to randomization varied from 3 to 14 days between the groups. The raw safety and efficacy outcomes of the trials are shown in the Data S1.

**Table 1:** Study characteristics

	Augustus	Re‐Dual PCI	Pioneer AF‐PCI	Entrust AF PCI
Patients (n)	2307	1527	1393	1506
Study design	P2Y12 inhibitor + Apixaban or VKA + aspirin or placebo for 6 months	Dual therapy with dabigatran (150 mg) +P2Y12 inhibitor or triple therapy with warfarin + aspirin +P2Y12 inhibitor for 12 months	Group 1: Rivaroxaban (15 mg) + SAPT (P2Y12 inhibitor) for 12 months inhibitor) Group 3: VKA + DAPT (aspirin + P2Y12 inhibitor)	Edoxaban + SAPT (P2Y12 inhibitor) for 12 months or VKA + DAPT (P2Y12 inhibitor + aspirin) for 1 to 12 months
Blinding	Placebo‐controlled	Open‐label	Open‐label	Open‐label
Time to randomization	14 days	5 days	3 days	5 days
Primary outcome	Major or CRNM bleeding at 6 months	Major or CRNM bleeding at 12 months	Clinically relevant bleeding at 12 months	Major or CRNM bleeding at 12 months
Treatment effect for intervention vs control	HR 0.53, 95% CI 0.45‐0.63, *P* < .001 for superiority	HR 0.72,95% CI 0.58‐0.88, *P* = .002 for superiority (dabigatran 150 mg bid)	HR 0.59, 95% CI 0.47‐0.76, *P* < .001 for superiority	HR 0.83, 95% CI 0.65‐1.05, *P* = .001 for noninferiority, *P* = .1154 for superiority
Year	2019	2017	2016	2019
Follow‐up	6 months	14 months	12 months	12 months

The Jadad scale for randomized controlled trials was used to determine the quality of the studies^26^ and is shown in the Data S1. Three of the four trials were open‐label, and blinding was not performed, except for AUGUSTUS. The outcomes were determined in a blinded manner and randomization was adequate.

### 3.3 Structure of the meta‐analysis

The study compared four treatment regimens comparing double therapy with DOACs vs triple therapy with VKAs: Apixaban + P2Y12 inhibitor vs VKA + DAPT; dabigatran 150 mg + P2Y12 inhibitor vs VKA + DAPT; low dose rivaroxaban 15 mg + P2Y12 inhibitor vs VKA + DAPT; edoxaban + P2Y12 inhibitor vs VKA + DAPT. Dabigatran 110 mg and low dose rivaroxaban of 2.5 mg were not analyzed. For the purpose of the analysis, we assumed that all of the DOACs at standard doses are equivalent and combined them for the meta‐analysis.

### 3.4 Patient‐centered outcomes

#### 3.4.1 ISTH major or clinically relevant nonmajor bleeding (CRNB)

**Figure 2:**
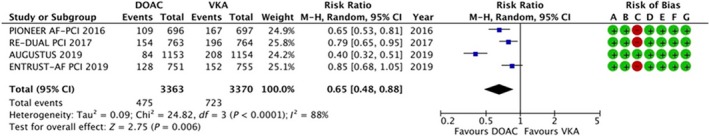
Forest plot and risk of bias analysis for DOAC vs VKA for ISTH major or clinically relevant nonmajor bleeding

The data were available for all the four trials, randomizing 6,733 patients. 1,198 of 6,733 patients experienced either ISTH major bleeding or CRNB. The forest plots of the analysis are shown in Figure 2. Results show that DOACs are associated with significantly lower bleeding compared to VKAs (RR: 0.65, 95% CI: 0.48‐0.88, *P* < .00001). A high degree of heterogeneity was noted (*I* ^2^ = 88%).

#### 3.4.2 All‐cause mortality

**Figure 3:**
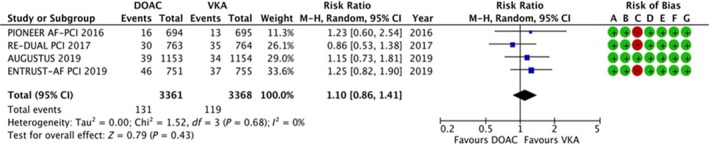
Forest plot and risk of bias analysis for DOAC vs VKA for all‐cause mortality

The data were available for all the four trails, reporting data for 6729 patients. Two hundred and fifty of 6729 patients had died. The results are summarized in Figure 3. There was no statistically significant difference in the risk of all‐cause mortality between double therapy with DOAC vs triple therapy with VKA (RR: 1.10, 95% CI: 0.86‐1.41, *P* = .43). No evidence of heterogeneity was noted (*I* ^2^ = 0%).

#### 3.4.3 Major adverse cardiovascular events (MACE)

**Figure 4:**
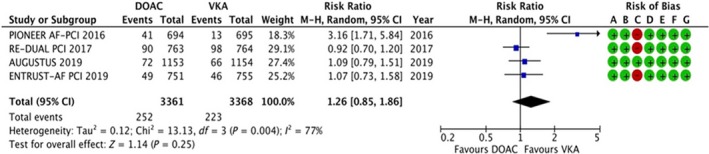
Forest plot and risk of bias analysis for DOAC vs VKA for major adverse cardiovascular event

The data were available for all the four trails, reporting data for 6729 patients. Four hundred and ninety‐eight of 6729 patients have experienced trial‐defined MACE. The results are summarized in Figure 4. There was no statistically significant difference in MACE (RR: 1.26, 95% CI: 0.85‐1.86, *P* = .25). A high degree of heterogeneity was noted (*I* ^2^ = 77%).

#### 3.4.4 Ischemic stroke

**Figure 5:**
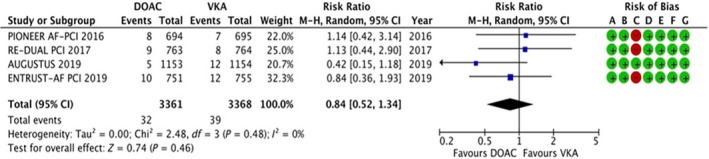
Forest plot and risk of bias analysis for DOAC vs VKA for ischemic stroke

The data were available for all the four trials, reporting data for 6729 patients. Seventy‐one of 6729 patients experienced a stroke, and the results are summarized in Figure 5. There was no statistically significant difference in the risk of stroke (RR: 0.84, 95% CI: 0.52‐1.34, *P* = .46). No evidence of heterogeneity was noted (*I* ^2^ = 0%).

#### 3.4.5 Myocardial infarction (MI)

**Figure 6:**
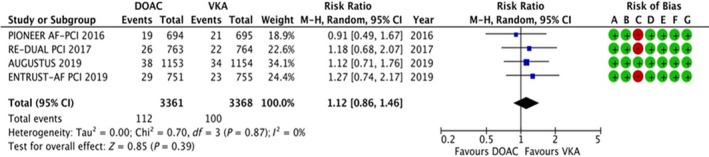
Forest plot and risk of bias analysis for DOAC vs VKA for myocardial infarction

The data were available for all the four trials, reporting data for 6729 patients. Two hundred and twelve of 6729 patients experienced an MI, and the results are summarized in Figure 6. There was no statistically significant difference in the risk of MI (RR: 1.12, 95% CI: 0.86‐1.46, *P* = .39). No evidence of heterogeneity was noted (*I* ^2^ = 0%).

#### 3.4.6 Stent thrombosis

**Figure 7:**
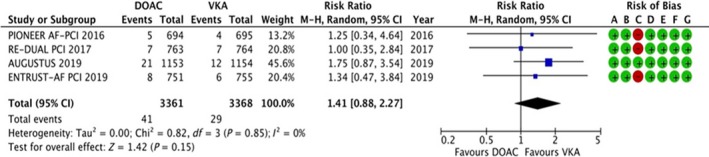
Forest plot and risk of bias analysis for DOAC vs VKA for stent thrombosis

The data were available for all the four trials, reporting data for 6,729 patients. Seventy of 6729 patients experienced stent thrombosis, and the results are summarized in Figure 7. There was no statistically significant difference in the risk of stent thrombosis (RR: 1.41, 95% CI: 0.88‐2.27, *P* = .15). No evidence of heterogeneity (*I* ^2^ = 0%).

## 4 DISCUSSION

In the present meta‐analysis of large RCTs, we showed a statistically significant reduced risk of ISTH major bleeding or clinically relevant nonmajor bleeding with no difference in all‐cause mortality, MACE, MI, ischemic stroke, or stent thrombosis in patients with AF who received double therapy with DOACs compared to standard triple therapy with VKAs following PCI.

The emergence of DOACS reshaped the anticoagulant therapy aspect of management of AF. The four RCTs included in this meta‐analysis are pivotal trials that address this important clinical scenario. RE‐DUAL PCI trial (Open‐Label, randomized, controlled, multicenter study), which evaluated double therapy with dabigatran 110 mg or 150 mg vs triple therapy with VKAs, showed that patients had noninferiority in respect of the risk of thromboembolic events, but the risk of bleeding was lower.^22^ The PIONEER AF‐PCI trial (Open‐Label, randomized, controlled, multicenter study) explored two treatment strategies of double therapy with rivaroxaban and triple therapy with VKA in patients with AF who underwent PCI and the results showed a lower rate of clinically significant bleeding with rivaroxaban treatments than with VKA and DAPT, with no significant difference in rates of ischemic events or major adverse cardiovascular events.^23^ The AUGUSTUS trial which was a prospective, multicenter, two‐by‐two factorial, and randomized clinical trial compared apixaban of 2.5 mg with a VKA and aspirin with placebo postacute coronary syndrome (ACS) or underwent PCI in patients with AF. The results showed that the antithrombotic regimen with apixaban had caused no significant differences in ischemic events but resulted in less bleeding and fewer hospitalizations when compared to a regimen including VKA, aspirin, or both.^24^ ENTRUST AF PCI (randomized, multicenter, open‐label trial) was performed to determine the safety of edoxaban plus P2Y12 inhibitor in patients with AF who had PCI and results showed that edoxaban‐based regimen was noninferior to VKA regimen for bleeding, without having significant differences in ischemic events.^25^

There is a delicate balance between the risk of bleeding and the risk of ischemia in patients with AF who develop ACS or undergo PCI. For these patients, an optimal regimen needs to be defined, with the primary considerations being double vs triple therapy and DOAC vs VKA. There are a range of studies and meta‐analyses on modifying regimens for anticoagulation in this patient population.^1^, ^7^ Moreover, trials have shown that triple therapy after PCI is associated with a twofold increase in bleeding in the patients relative to double therapy, and post‐PCI bleeding events are linked to worse MACE outcomes, possibly from the interruption in antithrombotic therapy during bleeding events.^28^, ^29^, ^30^, ^31^, ^32^ It has been shown that double therapy is enough for most patients with AF, and ACS/PCI and triple therapy may increase the risk of bleeding.^27^, ^33^, ^34^

We have shown that double therapy with DOACs is safe and as effective as triple therapy with VKA. These results favor the use of double therapy with a DOAC as the preferred therapeutic approach. In fact, these findings are highlighted in the recent 2019 ACC/AHA/HRS AF guidelines, in which double therapy with dabigatran or with rivaroxaban is recommended as first‐line therapy. Notably, these guidelines were published before the RCTs evaluating apixaban and edoxaban were published, and these two studies further solidify the role of double therapy with DOACs in patients with AF who underwent PCI. In the future, a dedicated RCT comparing each DOAC would provide valuable information regarding efficacy of different DOACs.

### 4.1 Summary of evidence

The present analysis updates the summary of evidence by adding two recent trials. Overall, the evidence is sufficiently strong to evaluate the comparative effectiveness of double therapy with DOACs and standard triple therapy with VKAs for patients with AF following PCI. The outcome with the highest grade of certainty of the evidence is ISTH major bleeding/ CRNB. The outcomes with moderate grade of certainty of the evidence include all‐cause mortality, MACE, stent thrombosis, myocardial infarction, and stroke (Table 2).

**Table 2:** Summary of evidence

Outcomes	Anticipated absolute effects^a^ (95% CI)		Relative effect (95% CI)	No. of participants (studies)	Certainty of the evidence (GRADE)^b^
	Risk with VKA	Risk with DOAC			
ISTH major or clinically relevant nonmajor bleeding	215 per 1000	142 per 1000 (127‐157)	RR 0.66 (0.59‐0.73)	6733 (4 RCTs)	⨁⨁⨁⨁ HIGH
All‐cause death	35 per 1000	39 per 1000 (31‐50)	RR 1.10 (0.87‐1.41)	6729 (4 RCTs)	⨁⨁⨁◯ MODERATE^c^
Major Adverse Cardiovascular events as defined by trials	66 per 1000	75 per 1000 (63‐89)	RR 1.13 (0.95‐1.35)	6729 (4 RCTs)	⨁⨁⨁◯ MODERATE^c^
Stroke	12 per 1000	9 per 1000 (6‐15)	RR 0.82 (0.52‐1.31)	6729 (4 RCTs)	⨁⨁⨁◯ MODERATE^c^
Myocardial infarction	30 per 1000	33 per 1000 (26‐43)	RR 1.12 (0.86‐1.46)	6729 (4 RCTs)	⨁⨁⨁◯ MODERATE^c^
Stent thrombosis	9 per 1000	12 per 1000 (8‐20)	RR 1.42 (0.88‐2.27)	6729 (4 RCTs)	⨁⨁⨁◯ MODERATE^c^

a The risk in the intervention group (and its 95% confidence interval) is based on the assumed risk in the comparison group and the relative effect of the intervention (and its 95% CI).

b GRADE Working Group grades of evidence: (a) High certainty: We are very confident that the true effect lies close to that of the estimate of the effect; (b) Moderate certainty: We are moderately confident in the effect estimate: The true effect is likely to be close to the estimate of the effect, but there is a possibility that it is substantially different; (c) Low certainty: Our confidence in the effect estimate is limited: The true effect may be substantially different from the estimate of the effect, (d) Very low certainty: We have very little confidence in the effect estimate: The true effect is likely to be substantially different from the estimate of effect

c Rated down for imprecision as the 95% confidence interval overlaps with no effect and fails to exclude important benefit or important harm.

### 4.2 Limitations

Follow‐up periods were different for the trials ranging from 6 to 12 months. DOACs and regimen used in the studies were different: Apixaban + P2Y12 inhibitor vs VKA + P2Y12 inhibitor; warfarin + P2Y12 inhibitor + aspirin vs dabigatran 150 mg + P2Y12 inhibitor; low dose rivaroxaban 15 mg + P2Y12 inhibitor vs VKA + DAPT; edoxaban + P2Y12 inhibitor vs VKA + P2Y12 inhibitor + aspirin. Interaction between several key groups such as type of index event, type of drug‐eluting stent, CHAD2S2‐VASc score, HAS‐BLED score could not be analyzed because of limited data in groups. There is significant heterogeneity between studies in terms of the design of the trial as well as the type and length of antiplatelet/antithrombotic therapy used, which could influence our result assessment. A small percentage of patients were lost to follow‐up. Quality of the studies varied as randomization was adequate. Given three of four were open‐label; overestimation of treatment effect in those trials was conceivable. The study population comprised predominantly of men, so there may be limited applicability of the findings of this study of female patients.

## 5 CONCLUSION

DOACs are associated with less risk of ISTH major/ CRNB bleeding and are as effective as standard therapy in patients with AF undergoing PCI.

## CONFLICT OF INTERESTS

The authors declare no conflict of interests for this article.

## AUTHORSHIP

All authors listed meet the authorship criteria according to the latest guidelines of the International Committee of Medical Journal Editors, and all authors are in agreement with the manuscript.
