# Supplementary material for: Safety and efficacy of direct oral anticoagulants compared to Vitamin K antagonists postpercutaneous coronary interventions in patients with atrial fibrillation: A systematic review and meta‐analysis
Source: J Arrhythm. 2020 Jan 8;36(2):271–9. doi: 10.1002/joa3.12292 (PMC7132188; doi:10.1002/joa3.12292)
Supplement: Supplementary file 1 [file JOA3-36-271-s001.docx]

**Supplementary Data**

**Contents**

1. Search strategy 2
2. Supplemental Tables

eTable 1: Efficacy outcomes of the included trials 3

eTable 2: Baseline characteristics of the subjects in the trials 4

eTable 3: Jadad scale to assess the quality of randomized control trials 9

1. Supplementary Figures

eFigure 1: Funnel plot- ISTH major/ clinically relevant non-major bleeding , 10

All-Cause Mortality and Major adverse cardiovascular event

eFigure 2: Funnel plot- Ischemic stroke, Myocardial Infarction and Stent 11

Thrombosis

**Search Strategy:**

**9.23.19 PubMed (9)**  ((((((dabigatran) OR dapt) OR rivaroxaban) OR clopidogrel) OR aspirin)) AND ((((("Warfarin"[Mesh]) OR Warfarin)) AND (("Atrial Fibrillation"[Mesh]) OR ("Atrial Fibrillation" OR AF))) AND ((("Percutaneous Coronary Intervention"[Mesh]) OR "Percutaneous Coronary Intervention") OR PCI)) Filters: Randomized Controlled Trial, English

**9.23.19 Web of Science (2)** TOPIC: ("atrial fibrillation" OR AF) AND TOPIC: (warfarin) AND TOPIC: (PCI OR "percutaneous coronary intervention") AND TOPIC: (RCT OR "randomly controlled trial")

Refined by: LANGUAGES: ( ENGLISH ) Indexes=SCI-EXPANDED, ESCI Timespan=All years

**9.23.19 ClinicalTrials.gov (45)** pci OR percutaneous coronary intervention | Atrial Fibrillation

**9.23.19 EBM Reviews - Cochrane Database of Systematic Reviews <2005 to September 11, 2019> Search Strategy (2)**

1 (dabigatran or rivaroxaban or clopidogrel or DAPT or aspirin).mp. [mp=title, short title, abstract, full text, keywords, caption text] (547)

2 warfarin.mp. [mp=title, short title, abstract, full text, keywords, caption text] (181)

3 (AF or atrial fibrillation).mp. [mp=title, short title, abstract, full text, keywords, caption text] (435)

4 (PCI or percutaneous coronary intervention).mp. [mp=title, short title, abstract, full text, keywords, caption text] (88)

5 1 and 2 and 3 and 4 (2)

**9.23.19 Author supplied** **(1)**

| DATABASE | RESULTS | DUPLICATES | REMAINING |
| --- | --- | --- | --- |
| PubMed | 9 | 0 | 9 |
| Web of Science | 2 | 0 | 2 |
| Cochrane Database of Systematic Reviews | 2 | 0 | 2 |
| ClinicalTrials.gov | 45 | 0 | 45 |
| Author Supplied | 1 | 0 | 1 |
| **TOTAL** | 59 | 0 | 59 |

**eTable 1:** Efficacy outcomes

|  | **Augustus** | | | | | | **Re-Dual** | | | | | | **Pioneer** | | | | | | **Entrust** | | | | | |
| --- | --- | --- | --- | --- | --- | --- | --- | --- | --- | --- | --- | --- | --- | --- | --- | --- | --- | --- | --- | --- | --- | --- | --- | --- |
|  | **Apixaban** | | **VKA** | | **p-value** | **HR** | **Dual therapy with dabigatran** | | **Triple therapy with warfarin** | | **p-value** | **HR** | **Low dose rivaroxaban** | | **VKA+ DAPT** | | **p-value** | **HR** | **Edoxaban** | | **VKA regimen** | | **p- value** | **HR** |
|  | **E** | **T** | **E** | **T** |  | **95% CI** | **E** | **T** | **E** | **T** |  | **95% CI** | **E** | **T** | **E** | **T** |  | **95% CI** | **E** | **T** | **E** | **T** |  | **95% CI** |
| **ISTH major/**  **CRNB** | 84 | 1153 | 208 | 1154 | 0.60 | 1.99 (1.563-2.60) | 154 | 763 | 196 | 764 | 0.002 | 0.72 (0.58-0.88) | 109 | 696 | 167 | 697 | <0.001 | 0.59 (0.47-0.76) | 128 | 751 | 152 | 755 |  | 0.85 (0.68-1.05) |
| **All-cause mortality** | 39 | 1153 | 34 | 1154 |  | 1.03 (0.75-1.42) | 30 | 763 | 35 | 764 | 0.44 | 0.83 (0.51-1.34) | 16 | 694 | 13 | 695 |  | 1.23 (0.60-2.54) | 46 | 751 | 37 | 755 | 0.3436 | 1.23 (0.8-1.897) |
| **MACE** | 72 | 1153 | 66 | 1154 |  | 1.09 (0.79-1.51) | 90 | 763 | 98 | 764 |  | 1.03 (0.84-1.25) | 41 | 694 | 36 | 695 | 0.75 | 1.08 (0.69-1.68) | 49 | 751 | 46 | 755 |  | 1.07 (0.73-1.58) |
| **Stroke** | 5 | 1153 | 12 | 1154 |  | 0.5 (0.29-0.97) | 9 | 763 | 8 | 764 | 0.85 | 1.09 (0.42-2.83) | 8 | 694 | 7 | 695 | 0.89 | 1.07 (0.39-2.96) | 10 | 751 | 12 | 755 | 0.678 | 0.84 (0.36-1.95) |
| **MI** | 38 | 1153 | 34 | 1154 |  | 0.89 (0.65-1.23) | 26 | 763 | 22 | 764 | 0.61 | 1.16 (0.66-2.04) | 19 | 694 | 21 | 695 | 0.62 | 0.86 (0.46-1.59) | 29 | 751 | 23 | 755 | 0.410 | 1.26 (0.73-2.17) |
| **Stent thrombosis** | 21 | 1153 | 12 | 1154 |  | 0.77 (0.38-1.56) | 7 | 763 | 7 | 764 | 0.98 | 0.99 (0.35-2.81) | 5 | 694 | 4 | 695 | 0.79 | 1.2 (0.32-4.45) | 8 | 751 | 6 | 755 | 0.600 | 1.32 (0.47-3.84) |

E- Events; T- Total; HR- Hazard Ratio; CI- Confidence Interval

**eTable 2:** Baseline Characteristics

| **Variables** | **Augustus** | | **Re-Dual PCI** | | **Pioneer PCI** | | **Entrust** | |
| --- | --- | --- | --- | --- | --- | --- | --- | --- |
|  | **Apixaban**  **(n=1153)** | **VKA**  **(n=1154)** | **Dabigatran 150 mg (n=763)** | **VKA**  **(n=764)** | **Rivaroxaban**  **(n=696)** | **VKA**  **(n=697)** | **Edoxaban**  **(n=751)** | **VKA**  **(n=755)** |
| **Age-median (Mean +/- SD)** | 69.8 +/- 9.31 | 70.5 +/-9.07 | 68.6+/-7.7 | 68.8+/-7.7 | 70.4+/-9.1 | 69.9+/-8.7 | 69.5 +/-4.1 | 70.2+/-3.7 |
| **Female sex n (%)** | 313  (27.14) | 339  (29.37) | 171 (22.4) | 170 (22.2) | 181 (25.5) | 188 (26.6) | 194 (26) | 192 (25) |
| **Elderly (>80 yrs;>70 yrs in Japan)** | |  | 8 (1) | 8 (1) |  |  |  |  |
| **>/=65 years of age n (%)** |  |  |  |  | 523 (73.8) | 526 (74.5) |  |  |
| **>/=75 years of age n (%)** |  |  |  |  | 254 (35.8) | 230 (32.6) |  |  |
| **Body weight** |  |  |  |  |  |  | 80 (71-93) | 83 (72-94) |
| **Race-n (%)** |  |  |  |  |  |  |  |  |
| **White** |  |  |  |  | 662 (93.4) | 664 (94.1) |  |  |
| **Black** |  |  |  |  | 7 (1.0) | 1 (0.1) |  |  |
| **Asian** |  |  |  |  | 25 (3.5) | 33 (4.7) |  |  |
| **Native American** |  |  |  |  |  |  |  |  |
| **American Indian or Alaska Native** | |  |  |  | 1 (0.1) | 0 (0) |  |  |
| **Other** |  |  |  |  | 14 (2.0) | 8 (1.1) |  |  |
| **Geographic region n (%)** |  |  |  |  |  |  |  |  |
| **Asia** |  |  |  |  |  |  | 82 (11) | 87 (12) |
| **Eastern Europe** |  |  |  |  |  |  | 350 (47) | 349 (46) |
| **Western Europe** |  |  |  |  |  |  | 319 (42) | 319 (42) |
| **Type of Atrial Fibrillation n (%)** | | | | | | | | |
| **Persistent** |  |  | 132 (17.3) | 149 (19.5) | 146 (20.6) | 149 (21.1) | 140 (19) | 146 (19) |
| **Permanent** |  |  | 250 (32.8) | 238 (31.2) | 262 (37.0) | 243 (34.5) | 209 (28) | 250 (33) |
| **Paroxysmal** |  |  | 380 (49.8) | 376 (49.3) | 300 (42.4) | 313 (44.4) | 402 (54) | 358 (47) |
| **Creatinine Clearance-ml/min** | 79.4 (31.7%) | 78.7 (30.2%) | 83.7 +/-31.0 | 81.3+/-29.6 | 78.3 +/-31.3 | 80.7 +/-30.0 | 72.1 +/-10.7 | 71.8 +/-10.3 |
| **Creatinine Clearance (30 - <60 ml/min)** | |  |  |  | 194 (28.8) | 175 (26.2) |  |  |
| **Creatinine Clearance(<30 ml/min)** | |  |  |  | 8 (1.2) | 2 (0.3) |  |  |
| **Index event n (%)** | | | | | | | | |
| **Acute Coronary Syndrome** |  |  | 391 (51.2) | 369 (48.3) | 216 (30.8) | 197 (28.5) |  |  |
| **Stable Angina or +ve stress test** |  |  | 320 (41.9) | 339 (44.4) |  |  |  |  |
| **Staged Procedure** |  |  | 138 (18.1) | 134 (17.5) |  |  |  |  |
| **Unstable Angina** |  |  |  |  | 145 (20.7) | 164 (23.7) |  |  |
| **Medical history n (%)** | | | | | | | | |
| **Myocardial Infarction** |  |  | 194 (25.4) | 211 (27.6) | 216 (27.9) | 197 (27.9) | 188 (25) | 177 (23) |
| **Previous PCI** |  |  | 239 (31.3) | 272 (35.6) |  |  | 199 (26) | 195 (26) |
| **Previous CABG** |  |  | 79 (10.4) | 87 (11.4) |  |  | 46 (6) | 49 (6) |
| **Congestive Heart Failure** | 483 (41.9) | 490 (42.5) |  |  | 180 (25.4) | 175 (24.8) | 418 (56) | 408 (56) |
| **Stroke** |  |  | 52 (6.8) | 77 (10.1) |  |  | 97 (13) | 92 (12) |
| **Peripheral Artery Disease** |  |  |  |  | 30 (4.2) | 35 (5) | 76 (10) | 82 (11) |
| **Diabetes Mellitus** | 414 (35.9) | 414 (35.9) | 260 (34.1) | 303 (39.7) | 204 (28.8) | 221 (31.3) | 259 (34) | 528 (34) |
| **Hypertension** | 1024 (88.8) | 1013 (87.8) |  |  | 520 (73.3) | 532 (75.4) | 674 (90) | 687 (91) |
| **Hypercholesterolemia** |  |  |  |  | 302 (42.6) | 316 (44.8) | 497 (66) | 484 (64) |
| **Bleeding events** |  |  |  |  |  |  | 56 (7) | 49 (6) |
| **Valvular heart disease** |  |  |  |  |  |  | 210 (28) | 221 (29) |
| **Malignancy** |  |  |  |  |  |  | 43 (6) | 46 (6) |
| **Non CNS systemic embolic event** | |  |  |  |  |  | 12 (2) | 10(1) |
| **Stroke/TIA/Thromboembolism** | 326 (14.2) | 307 (13.4) |  |  |  |  |  |  |
| **HAS-BLED Score** |  |  | 2.6+/-0.7 | 2.7 +/-0.8 |  |  | 2.75 +/-0.14 | 2.75 +/-0.14 |
| **Current smoker n (%)** |  |  |  |  | 37 (5.2) | 48 (6.8) |  |  |
| **Type of stent n (%)** |  |  |  |  |  |  |  |  |
| **Drug eluting** |  |  | 621 (81.5) | 638 (84.1) | 464 (65.4) | 468 (66.5) |  |  |
| **Bare metal** |  |  | 123 (16.1) | 107 (14.1) | 231 (32.6) | 224 (31.8) |  |  |
| **Drug eluting and bare metal** |  |  | 10 (1.3) | 9 (1.2) | 14 (2) | 12 (1.7) |  |  |
| **Other** |  |  | 8 (1) | 5 (0.7) |  |  |  |  |
| **Clopidogrel** |  |  |  |  | 660 (93.1) | 680 (96.3) | 696 (93) | 695 (92) |
| **Prasugrel 5 mg** |  |  |  |  |  |  | 2 (<1) | 1 (<1) |
| **Prasugrel 10 mg** |  |  |  |  | 12 (1.7) | 5 (0.7) | 3 (<1) | 2 (<1) |
| **Ticagrelor** |  |  |  |  | 37 (5.2) | 21 (3) | 49 (7) | 57 (8) |
|  |  |  |  |  |  |  |  |  |
| **No.of days from ACS or PCI to randomization** |  |  |  |  |  |  | 47.02+/-15.3 | 47.05+/-15.3 |
| **CHA2DS2-VASc score** | 3.9 +/-1.6 | 4 +/-0.6 | 3.3 +/-1.5 | 3.6 +/-1.5 | 0: 11 (1.6) | 7 (1) | 4 +/-0.61 | 4 +/-0.61 |
|  |  |  |  |  | 1: 66 (9.3) | 44 (6.2) |  |  |
|  |  |  |  |  | 2:112 (15.8) | 96 (13.6) |  |  |
|  |  |  |  |  | 3: 125 (17.6) | 148 (21) |  |  |
|  |  |  |  |  | 4:138 (19.5) | 174 (24.6) |  |  |
|  |  |  |  |  | 5: 140 (19.7) | 125 (17.7) |  |  |
|  |  |  |  |  | 6: 93 (13.1) | 91 (12.9) |  |  |
|  |  |  |  |  | 7: 24 (3.4) | 21 (3) |  |  |
| **Type of therapy before PCI n (%)** |  |  |  |  |  |  |  |  |
| **VKA** |  |  |  |  |  |  | 232 (31) | 224 (30) |
| **NOAC** |  |  |  |  |  |  | 176 (23) | 189 (25) |
| **None** |  |  |  |  |  |  | 192 (26) | 221 (29) |
| **Missing data** |  |  |  |  |  |  | 151 (20) | 121 (16) |

**eTable 4:** Quality of randomized clinical trials assessed using Jadad scale

| **Study** | **Was the study described as random?** | **Was the randomization scheme described and appropriate?** | **Was the study described double- blinded?** | **Was the method of double blinding appropriate?** | **Was there a description of dropouts and withdrawals?** | **Score** |
| --- | --- | --- | --- | --- | --- | --- |
| Augustus | 1 | 1 | 1 | 1 | 1 | 5 |
| Re-Dual | 1 | 1 | 0 | N/A | 1 | 3 |
| Pioneer | 1 | 1 | 0 | N/A | 1 | 3 |
| Entrust | 1 | 1 | 0 | N/A | 1 | 3 |


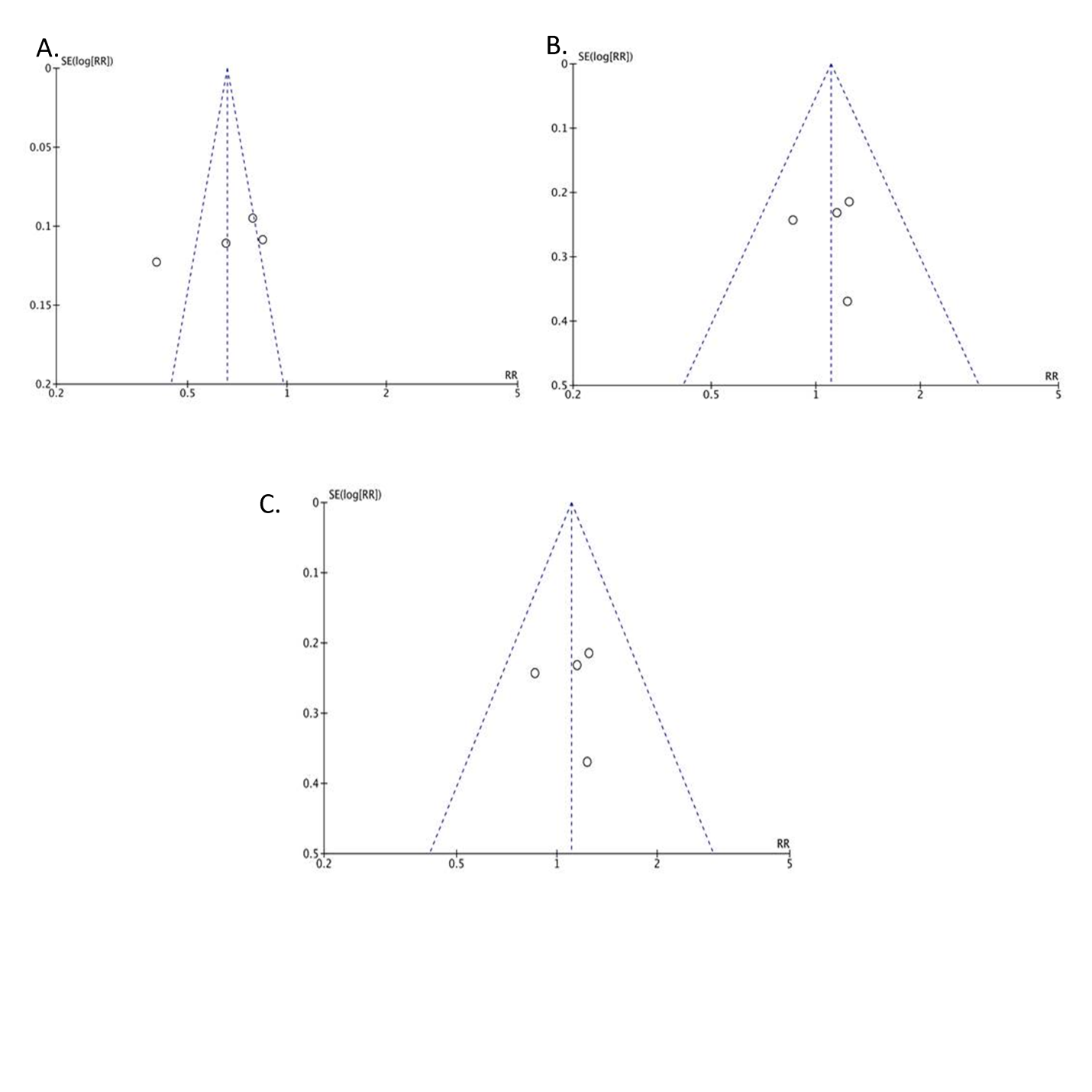


**eFigure 1:** Funnel plot: A) ISTH major / clinically relevant non-major bleeding B) All-Cause Mortality C) Major adverse cardiovascular events


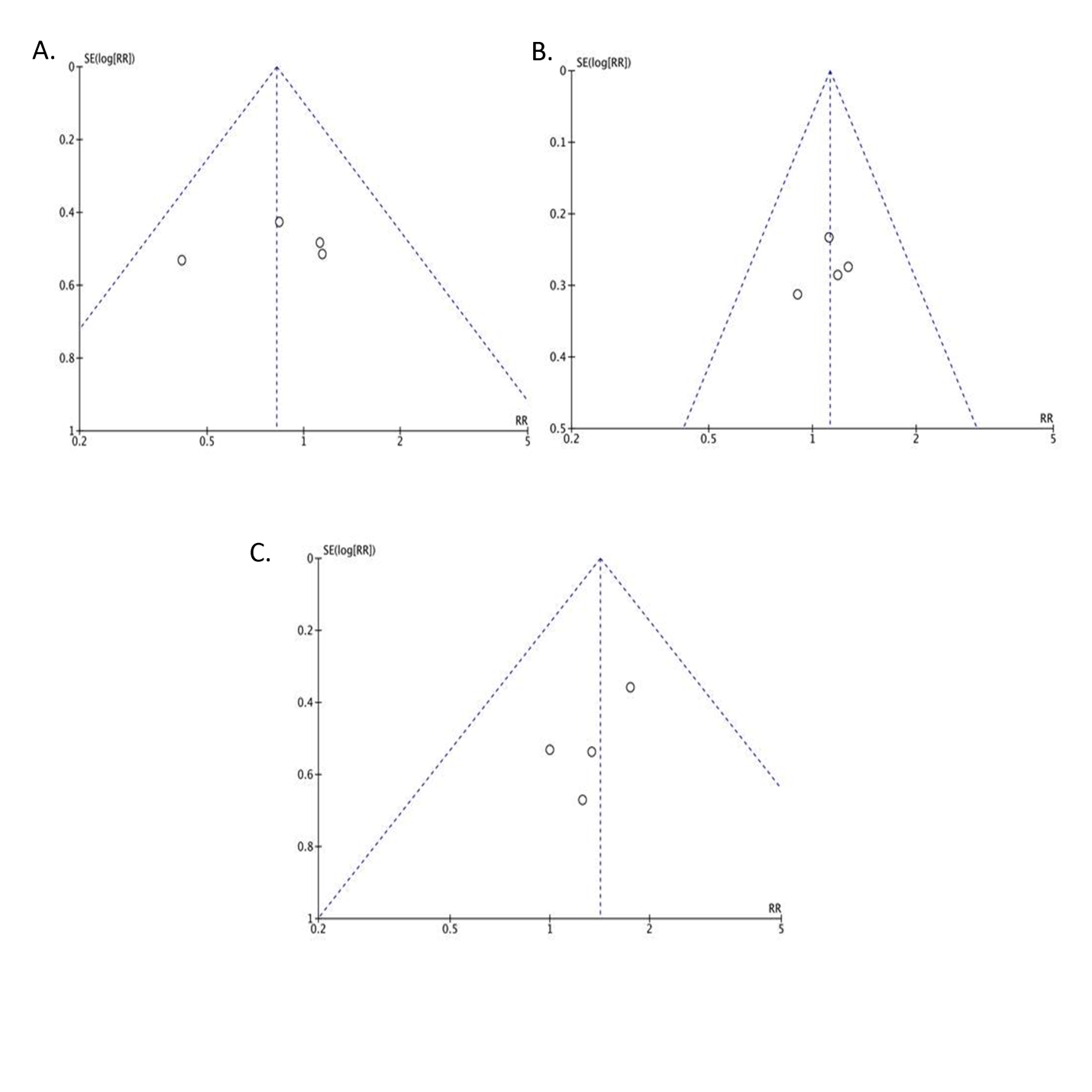


**eFigure 2**: Funnel plots: A) Ischemic Stroke B) Myocardial Infarction C) Stent Thrombosis
